# The association of neurodevelopmental abnormalities, congenital heart and renal defects in a tuberous sclerosis complex patient cohort

**DOI:** 10.1186/s12916-022-02325-0

**Published:** 2022-04-20

**Authors:** Jessica Robinson, Orhan Uzun, Ne Ron Loh, Isabelle Rose Harris, Thomas E. Woolley, Adrian J. Harwood, Jennifer Frances Gardner, Yasir Ahmed Syed

**Affiliations:** 1 Neuroscience and Mental Health Research Institute, Hadyn Ellis Building, Cardiff, CF24 4HQ UK; 2 School of Bioscience, The Sir Martin Evans Building, Museum Ave, Cardiff, CF10 3AX UK; 3 University Hospital of Wales, Heath Park, Cardiff, CF10 3AX UK; 4 Royal United Hospitals Bath NHS Foundation Trust, Bath, BA1 3NG UK; 5 School of Mathematics, Cardiff University, Cardiff, CF24 4AG UK

**Keywords:** TSC1, TSC2, Rhabdomyoma, Neurodevelopmental disorders, TAND, Kidney lesions

## Abstract

**Background:**

Tuberous sclerosis complex (TSC) is a rare multi-system genetic disorder characterised by the presence of benign tumours throughout multiple organs including the brain, kidneys, heart, liver, eyes, lungs and skin, in addition to neurological and neuropsychiatric complications. Intracardiac tumour (rhabdomyoma), neurodevelopmental disorders (NDDs) and kidney disorders (KD) are common manifestations of TSC and have been linked with *TSC1* and *TSC2* loss-of-function mutations independently, but the dynamic relationship between these organ manifestations remains unexplored. Therefore, this study aims to characterise the nature of the relationship specifically between these three organs’ manifestations in *TSC1* and *TSC2* mutation patients.

**Methods:**

Clinical data gathered from TSC patients across South Wales registered with Cardiff and Vale University Health Board (CAV UHB) between 1990 and 2020 were analysed retrospectively to evaluate abnormalities in the heart, brain and kidney development. TSC-related abnormalities such as tumour prevalence, location and size were analysed for each organ in addition to neuropsychiatric involvement and were compared between *TSC1* and *TSC2* mutant genotypes. Lastly, statistical co-occurrence between organ manifestations co-morbidity was quantified, and trajectories of disease progression throughout organs were modelled.

**Results:**

This study found a significantly greater mutational frequency at the *TSC2* locus in the cohort in comparison to TSC1. An equal proportion of male and female patients were observed in this group and by meta-analysis of previous studies. No significant difference in characterisation of heart involvement was observed between *TSC1* and *TSC2* patients. Brain involvement was seen with increased severity in *TSC2* patients, characterised by a greater prevalence of cortical tubers and communication disorders. Renal pathology was further enhanced in *TSC2* patients, marked by increased bilateral angiomyolipoma prevalence. Furthermore, co-occurrence of NDDs and KDs was the most positively correlated out of investigated manifestations, regardless of genotype. Analysis of disease trajectories revealed a more diverse clinical outcome for *TSC2* patients: however, a chronological association of rhabdomyoma, NDD and KD was most frequently observed for *TSC1* patients.

**Conclusions:**

This study marks the first empirical investigation of the co-morbidity between congenital heart defects (CHD), NDDs, and KDs in *TSC1* and *TSC2* patients. This remains a unique first step towards the characterisation of the dynamic role between genetics, heart function, brain function and kidney function during the early development in the context of TSC.

**Supplementary Information:**

The online version contains supplementary material available at 10.1186/s12916-022-02325-0.

## Background

Tuberous sclerosis complex (also known as tuberous sclerosis or TSC) is a multi-system autosomal dominant neurocutaneous genetic disorder prevalent in 1 in 6000 to 1 in 12,000 live births [1]. The disease results from a variety of loss-of-function mutations in the tumour suppressor genes *TSC1* and *TSC2*, with more than 200 *TSC1* and nearly 700 *TSC2* unique allelic variants having been identified in TSC patients thus far [2, 3]. The condition is classified as tuberous sclerosis complex 1 or 2 depending on whether their mutation falls within the respective *TSC1* or *TSC2* genetic loci. However, genetic testing has revealed *TSC2* mutations account for an overwhelming 70–90% of cases, which are often correlated with a more severe clinical outcome [2, 4–7]. TSC presents diverse clinical manifestations throughout multiple organ systems, most notably hamartomas (a type of benign tumour) in the brain, heart, kidneys, skin and lungs [8]. Additionally, 90% of TSC patients also suffer from TSC-associated neuropsychiatric disorders (TAND), an umbrella term describing the range of neurodevelopmental (NDD), psychiatric, psychosocial and behavioural disorders which vary from patient to patient [9, 10]. TSC is a highly complex and variable disease; however, it has been reported that fewer than half of individuals with TSC receive adequate centralised coordinated care in adherence to TSC patient management guidelines [11]. In light of this, it is imperative that all TSC patients receive coordinated care and that this care is supported by management guidelines that reflect up to date research and accounts for the co-morbidity of TSC manifestations, rather than reviewing each on an independent basis.

Cardiac rhabdomyoma (CR) accounts for 45% of primary cardiac tumours in children, making it the most common form of childhood cardiac tumour [12, 13]. Approximately 70–90% of children with CRs will also be diagnosed with TSC and conversely 90% of children with TSC under 2 years old will either have single or multiple CRs [12, 14]. CRs are a variety of benign mesenchymal tumour composed of cardiac myocytes, categorised within a group of late-onset congenital heart diseases [15]. Rhabdomyoma can appear as a singular or multiple tumours and are able to develop in all myocardial areas, although are most frequently located within the septal or ventricular walls wherein they can range in size from only a few millimetres to several centimetres [12, 16]. Although benign in histology, a minority of patients with CRs may experience symptoms before and shortly after birth which could include arrhythmias and/or obstruction of inflow or outflow by tumours which may lead to ventricular dysfunction and ultimately heart failure [12, 15]. Multiple congenital CRs are a well-established early marker for TSC and are often one of the first warning signs that lead to a diagnosis, appearing prenatally at around 20–30 weeks’ gestation and detectable by foetal echocardiography, which is now the primary diagnostic tool for paediatric cardiac tumour evaluation [17, 18]. Typically, spontaneous regression of CRs occurs within the first year of life for the majority of TSC patients, with prevalence decreasing to approximately 20% once the patient reaches 2 years old [8, 12].

TSC manifests in the brain as anatomical abnormalities and other neurological complications such as seizures, cognitive impairment and behavioural concerns. Around 90% of TSC patients exhibit some form of classic TSC brain pathology including: cortical tubers, subependymal nodules (SEN), subependymal giant cell astrocytoma (SEGA) and white matter migration tracts [19].

Previous studies have observed these brain lesions as early as 20 weeks of gestation in the developing foetal brain [20]. The most common brain manifestations observed in TSC are cortical tubers (glioneuronal hamartomas), small regions of cerebral cortical dysplasia that appear during early brain development and display impaired or absent cortical lamination as well as the presence of dysmorphic neurons, and can range from only a few millimetres to several centimetres in size [21, 22]. Unlike cortical tubers, SEN and SEGA are benign tumours composed of glial neuroastrocytes and tend to form deeper within the brain, particularly along the ependymal lining of the ventricles [23].

Approximately, 80% of TSC patients develop SEN, which are most commonly located near the caudate nucleus behind the foramen of Monro, where they usually appear in their multiples, with singular lesions ranging from 2 to 10mm in diameter and remaining stable in size in most cases [24, 25]. Whilst the majority of SEN cases are asymptomatic, 5–10% of cases degenerate into SEGA which can grow large enough to obstruct the foramen of Monro resulting in intracranial hypertension, blindness or death if untreated [26].

In addition to anatomical abnormalities in neurology, epilepsy is a further major cause of mortality and morbidity in TSC patients, with prevalence reported to range from 63 to 93% [27]. Epilepsy and infantile spasm associated with TSC first become apparent usually within the first year of life and has a strong association with both neurodevelopmental and cognitive problems [28]. TSC is also associated with a diverse range of cognitive, behavioural and psychiatric manifestations which are referred to as TSC-associated neuropsychiatric disorders (TAND) by specialists.

TAND can be investigated at six levels: behavioural, psychiatric, intellectual, academic, neuropsychological and psychosocial [10]. In total, it is estimated that around 90% of TSC individuals will experience some features consistent with TAND over their lifetime [29]. Many disorders under the TAND umbrella also overlap with the Diagnostic and Statistical Manual of Mental Disorders, 5th Edition (DSM-5) classification of neurodevelopmental disorders. Such disorders within this category include intellectual disabilities (both intellectual disability (ID) and global/developmental delay (GDD), communication disorders, autism spectrum disorder (ASD), attention-deficit/hyperactivity disorder (ADHD) and motor disorders [30]. The most frequent NDDs burdening TSC patients are ID and ASD, which affect 50% of individuals and share common risk factors including the presence of *TSC2* mutation, structural brain abnormalities and epilepsy [31].

Renal manifestations of TSC are the second most observed symptom of the disease after brain manifestations, with renal angiomyolipoma (AMLs) and renal cystic disease present in 80% and 30% of individuals, respectively [32, 33]. Renal AMLs are the most common type of benign renal tumour and have a prevalence of 0.2–0.6% in the general population, with 20% of these cases being associated with TSC [34]. These tumours are classified as a heterogenous group of neoplasms and are composed of three elements in variable amounts; blood vessels (angio-), smooth muscle (myo-) and adipose tissue (lipo-) [35]. Renal AMLs are present in approximately 75% of TSC patients and whilst the majority of cases remain asymptomatic when tumours are small, larger tumours may result in loin pain, catastrophic haematuria and hypertension [34, 36]. Renal cysts are a secondary manifestation of TSC in the kidneys, however are observed with less frequency than AMLs [32]. Cysts exhibit two modes of presentation: the most common being single or multiple lesions that are rarely symptomatic and histologically uniform, and the other presentation involving large, symptomatic cysts as a result of polycystic kidney disease (PKD), which is linked to deletions of *TSC2* with contiguous deletion of adjacent *PKD1* [37].

**Fig. 1:**
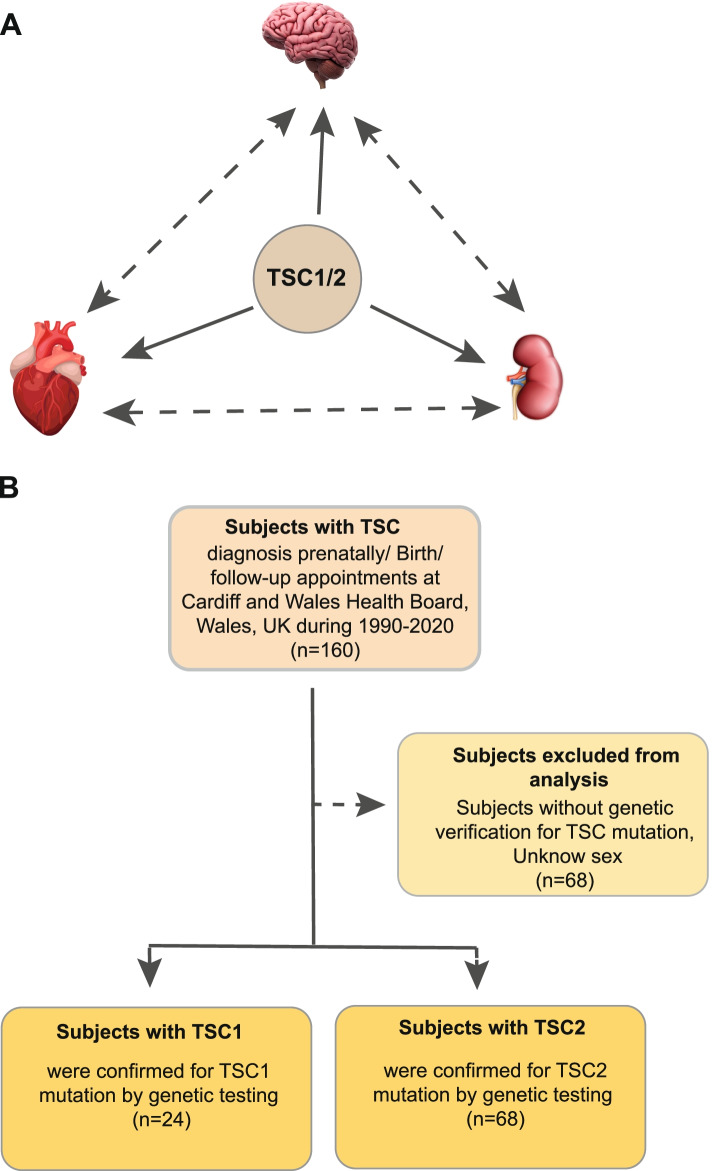
Graphical abstract and flow chart of the study depicting exclusion criteria for data analysis during this investigation. **A** The aim of the study was to look at association of comorbidities of the tuberous sclerosis complex (TSC1/2) on clinical organ manifestations of the heart, brain and kidney using the TSC patient cohort of 30 years referred to Cardiff and Vale University Health Board (CAV UHB). **B** Raw data from 160 patients with TSC provided by CAV UHB was filtered according to outlined exclusion criteria to improve cohort suitability for data analysis. Patients were separated into either TSC1 group or TSC2 group depending on results from genetic testing, and respective sample sizes are summarised. TSC, tuberous sclerosis complex; CAV UHB, Cardiff and Vale University Health Board

This study seeks to systematically evaluate abnormalities in the heart, brain and kidney development in patients with *TSC1* and *TSC2* mutations and establish an association between the three as no other study has done before (Fig. 1A). These three organ complications were selected initially as they are the most commonly investigated manifestation. Correlation between TSC gene mutations and incidence of CR, kidney disorders and poorer neurodevelopmental outcomes in children have long since been investigated independently of one another, but few studies have attempted to establish a direct link between two of these variables, let alone three.

To achieve this, a retrospective clinical study was conducted using data from TSC patient databases at Cardiff and Vale University Health Board (CAV UHB). This TSC lifespan centre is the only referral clinic for South Wales and the only hospital performing TSC genetic testing for the 30-year study period. Using this cohort, a breakdown of comorbidity between CR, NDDs and KDs in both *TSC1* and *TSC2* patients will be presented which will provide a clear genotype-phenotype profile of these three organ manifestations of TSC. The outcomes of this study may inform future clinical recommendations for TSC patient management for comorbidity between CR, NDDs and RDs and direct future clinical strategy which considers comorbidity of these three manifestations as a direct link of one another instead of being treated as independent disorders.

## Methods

### Data collection

This investigation is a retrospective cohort study of clinical data from TSC patients that has been collected by Cardiff and Vale University Health Board (CAV UHB) over a 30-year period. The clinical patient database used includes 160 anonymous patients situated all across South Wales who were referred to the hospital and fulfilled the current TSC clinical diagnostic criteria either prenatally, at birth, or at follow-up appointments between 1990 and up until the end of 2020 [38]. This database of registered patients with TSC uses multiple sources of patient data ascertainment which includes patient diagnostic data from multiple hospital departments such as the Institute of Clinical Genetics, Paediatric Cardiology, Neurology, Renal Unit, Dermatology, Ophthalmology and Respiratory medicine. Genetic data was logged in accordance with standard nomenclature recommendations by the Human Genome Variation Society (HGVS) as used in practice by the Institute of Medical Genetics in Cardiff. NDD data was also recorded and standardised in line with the Diagnostic and Statistical Manual of Mental Disorders, 5th Edition (DSM-5) classifications of NDDs; however, this is likely to be incomplete due to a frequently observed treatment and assessment gap of TAND in the UK [29]. Lastly, cardiac, renal and neurological data were collected in a more generalised standard format, with a general descriptor such as ‘rhabdomyoma’ for cardiac information, ‘AML’ or ‘cyst’ for renal information and ‘cortical tuber’, ‘SEN’, ‘SEGA’ or ‘epilepsy’ for neurological information.

Ethical approval of this retrospective review study was not necessary as the data was retrieved anonymously from the previously collected clinical information in various databases. Nevertheless, this study was approved as an audit project by the Cardiff and Vale University Health Board and registered on the Clinical Audit Database, reference no: 9618.

### Identification of genetic abnormalities

Genetic data of specific TSC mutations was kindly provided by the Institute of Medical Genetics in Cardiff. Data was obtained either via previous research testing or attendance of patients at the resident TSC clinic, and records and results of these tests are held on the Medical Genetics departmental system, Shire. Historically, the Institute of Medical Genetics employed Sanger sequencing (SS) methods to identify mutations during genetic testing; however, this has since evolved in favour of the next-generation sequencing (NGS) technology. Identification of *TSC1/2* mutations is a vital step towards establishing a clear clinical diagnosis of TSC, with the recommendation of the 2012 and updated 2021 International TSC Consensus Conference suggesting that identification of a pathogenic *TSC1* or *TSC2* mutation should be sufficient for the diagnosis or prediction of TSC regardless of clinical findings [38]. With this recommendation in mind, an exclusion criterion was proposed where in order for a patient to be included in data analysis they must have been genetically tested and identified with a *TSC1* or *TSC2* mutation in addition to known sex to improve the quality of data records and improve the suitability of the cohort for data analysis (Fig. 1B).

All genetic alterations were annotated and reported in accordance with recommendations of the HUGO Gene Nomenclature Committee (https://www.genenames.org/; http://www.hgvs.org/). Single-nucleotide variants (SNVs) and insertion-deletion mutations (indels) were reported using standard mutation nomenclature based on coding DNA reference sequences or protein-level amino acid sequences which require the prefixes of ‘c.’ or ‘p.’, respectively [39]. Exonic sequences are numbered according to their sequential position from the initiation codon to the stop codon and intronic sequences are also numbered in relation to the exonic coding sequence. Each record was then grouped into one of several mutation type descriptions; nonsense, missense, intronic (missense), frameshift, large deletion or ‘unknown’ when there were no further details of genetic mutation besides simply ‘TSC1 mutation’ or ‘TSC2 mutation’.

### Mathematical modelling

In order to evaluate the association between organ systems in the context of TSC and provide probabilities of possible disease trajectories throughout life, mathematical modelling techniques were explored in collaboration with Cardiff University School of Mathematics and employed in this study. Comorbidity data was provided for each patient in addition to the age at which each organ system manifestations of TSC was first detected in the patient. The terminology was specified where three outcomes were defined; *CHD*, where a patient presents rhabdomyoma; *NDD*, where a patient has one or more neurodevelopmental disorders; and *KD*, where a patient presents with kidney lesions. Co-occurrence was denoted in Boolean operator terms as the ‘cap’ notation visually represented by ‘∩’, so for example where *CHD* denotes the presence of a CHD, *NDD* denotes NDD and *KD* denotes renal involvement and *CHD* ∩ *NDD* would represent a scenario where a patient has both a CHD and an NDD. Using temporal data, supposing we know where organ manifestations start originally, the aim is to predict where they will appear next for *TSC1* and *TSC2* patients separately. To do this, all the possible trajectories and their probabilities were enumerated from the data. Namely, where were manifestations of TSC first detected (i.e. heart, *CHD*; brain, *NDD*; or kidneys, *KD*) and where will they transition next? Probabilities of all possible disease trajectories for this cohort are defined in Tables S1 and S2.

### Statistical analysis

All statistical analysis of the data was performed in GraphPad Prism version 9.1 (GraphPad Software, San Diego, California, USA, www.graphpad.com) and Microsoft Excel. Two-sided *p* values of *p* < 0.05 were considered statistically significant. Where applicable, data was assessed for Gaussian distribution with Shapiro-Wilk test and generated QQ plots of predicted vs actual data are found in Supplementary information (Additional file 1: Figs. S1, S6, S7 and S10). Due to the nature of data collection for this project, it is important to note that no control group information (i.e. a group with no *TSC1/2* mutation) is included within this data set. When comparing *TSC1* vs *TSC2* patient group prevalence within one categorical variable (i.e. mutation type, rhabdomyoma prevalence), Fisher’s exact test was used to assess statistical significance when the overall sample size was 30 patients or fewer, and alternatively for larger sample sizes, a *Z*-score test for two population proportions was used.

Cochrane’s *Q* test for heterogeneity was employed to assess heterogeneity between male to female ratio (M:F) during meta-analysis of previous studies assuming a null hypothesis that the true effect is the same across all studies and variations are due to statistical chance alone. A more powerful quantity *I*^2^ is derived from the *Q*-statistic by dividing the resulting statistic and its degrees of freedom by the *Q* value and was also used to provide an estimate of the percentage in variability across studies due to factors other than chance. Logarithmic trendlines for graphs of the rate of rhabdomyoma regression, rate of brain involvement detection and rate of kidney involvement detection were generated in Microsoft Excel along with the *R*^2^ coefficient of determination to assess the goodness of fit of the trendline to the fitted values and observed values. Residual plots of fitted values vs residuals are included in Supplementary information (Additional file 1: Figs. S2-S5, S8 and S9). Two-tailed Wilcoxon matched-pairs signed-rank tests were also performed between TSC1 and TSC2 groups to assess whether their population mean ranks differ. Significant effectiveness of pairings was assessed with Spearman’s rank, rs.

## Results

### Burden and characteristic features of TSC1 and TSC2 in a cohort

In order to fulfil the aims of this study, the characterisation of the patient cohort was first conducted. To do this, examination of several key variables was necessary which included the prevalence and type of each specific TSC mutation, ratio of males to females, and diagnostic data of the brain, kidney, and heart to further delve into the very essence of TSC as a multisystem heterogenous genetic disorder.

**Fig. 2:**
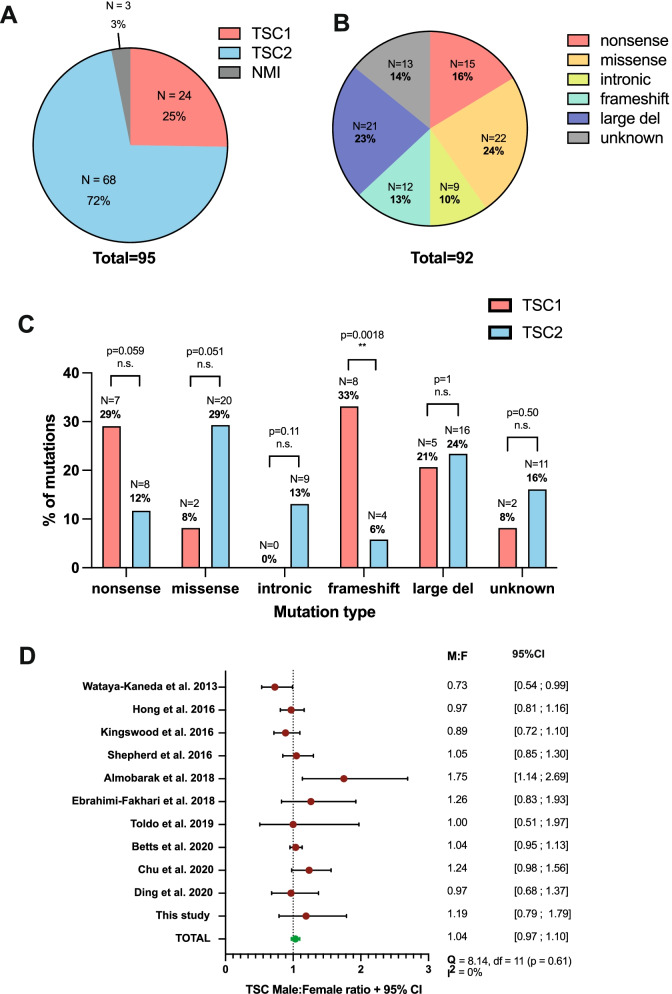
Genetic characterisation and mutational spectra of pathogenic TSC mutations and male to female ratio. **A** Number and proportion of cases with TSC1 or TSC2 mutation or no mutation identified (NMI); **B** Number and prevalence of mutation types of 92 patients with an identified genetic mutation; **C** Proportions of each mutation type in TSC1 vs TSC2 compared with Fisher’s exact test (*p* < 0.05 significance); **D** Forest plot indicating the male to female ratio calculated from each study involving a TSC patient cohort (red circle), and the overall mean male to female ratio is represented by the green circle, confidence whiskers represent a 95% confidence interval (CI) for each study and the mean, male to female ratio (M:F) and upper and lower confidence intervals are listed in right hand columns where a M:F of 1 (*x* = 1) signifies equal number of males and females in each cohort as graphically indicated by the dotted line, *x*<1 represents more females than males and *x*>1 represents more males than females, Cochran’s *Q* test for heterogeneity: *Q*=8.14, df=11 (*p*=0.61); *I*^2^=0%; CI, confidence interval; TSC, tuberous sclerosis complex; ** *p* < 0.01. ; n.s., non-significant

**Fig. 3:**
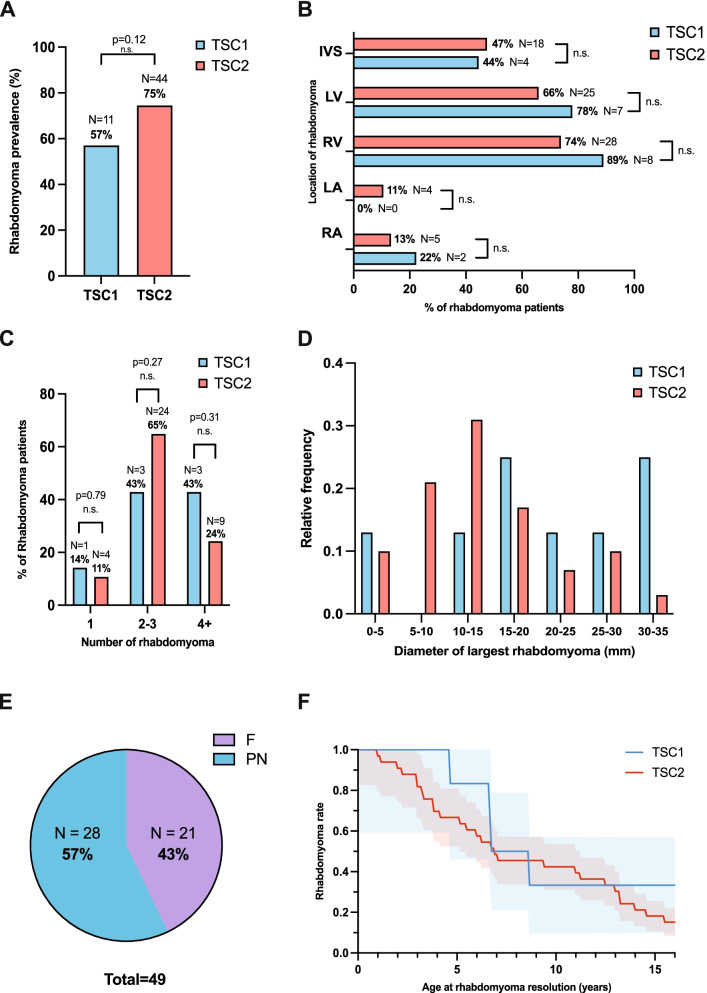
Characterisation of cardiac rhabdomyoma in TSC1 and TSC2 patients by echocardiography. **A** The prevalence of cardiac rhabdomyoma in TSC1 vs TSC2 patients (TSC1, *N* = 11; TSC2, *N* = 44); **B** The location of cardiac rhabdomyoma within heart chambers of TSC1 vs TSC2 rhabdomyoma patients (TSC1, *N* = 9; TSC2, *N* = 38); **C** The number of rhabdomyoma in hearts of TSC1 vs TSC2 rhabdomyoma patients (TSC1, *N* = 7; TSC2, *N* = 37); **D** The size of the largest rhabdomyoma in TSC1 vs TSC2 patients (TSC1, *N* = 8; TSC2, *N* = 29); **E** The proportion of patients diagnosed with rhabdomyoma as a foetus (F) vs proportion of postnatally (PN) diagnosed patients (*N* = 49); **F** The rate of the rhabdomyoma prevalence as tumours resolve in TSC1 vs TSC2 rhabdomyoma patients (TSC1, *N* = 7; TSC2, *N* = 33) as age increases (mean ± SEM); TSC1 vs TSC2 statistically compared with *Z*-score for 2 population proportions (*p* < 0.05 significance); IVS, interventricular septum; LV, left ventricle; RV, right ventricle; LA, left atrium; RA, right atrium; n.s., non-significant; SEM, standard error of mean; *p < 0.05

*TSC1* and *TSC2* mutations in the patient cohort were identified using NGS and SS methods. *TSC2* variants represented the majority of the cohort, constituting approximately 72% (*N* = 68) of all 95 genetically tested TSC patients (Fig. 2A). In contrast, only 25% of tested patients had *TSC1* mutations (*N* = 24), and no mutation was identified (NMI) in the remainder (*N* = 3) (Fig. 2A). This means that the ratio of TSC1 to TSC2 patients for this cohort is approximately 1:2.8. A further breakdown of the mutation variant types of the 92 patients with an identified mutation is summarised in Fig. 2B, where 63% of all changes corresponded to small variants (SV) such as small deletions or insertions, duplications, or point mutations. There was also a further group of 13 patients, where no mutation data was available on hospital databases beyond unspecified ‘TSC1 mutation’ or ‘TSC2 mutation’, who were therefore classed into an ‘unknown’ category. The data presented in Fig. 3B was then further explored in Fig. 2C where the proportion of each type of variant in *TSC1* vs *TSC2* was compared and statistically evaluated. There was no significant difference between *TSC1* and *TSC2* groups in the case of nonsense mutations (*p* = 0.059), missense mutations (*p* = 0.051), intronic mutations (*p* = 0.105) and large deletions (*p* = 1.00). However, a significant difference in the proportion of frameshift mutations was identified (*p* = 0.0018) comprising 33% and 6% of the *TSC1* vs *TSC2* groups, respectively.

Next, a meta-analysis was conducted of the male to female ratio (M:F) for this study in addition to ten other previous studies of TSC patient cohorts (Fig. 2D). Within the CAV UHB cohort, there were 50 male patients and 42 females, resulting in a M:F ratio of 1.19:1 (Fig. 3D). The 95% confidence interval (CI) intersects with the line of no effect (*x* = 1), and thus, at the given level of confidence, the M:F ratio does not differ from equal ratio and is therefore statistically insignificant (Fig. 3D). Explicitly, TSC burdens male and female patients equally in this particular cohort. Out of the other ten studies inspected, a further eight were also synonymous with this result and also showed equal burden of TSC in male and female patients. The total M:F ratio across all studies was calculated at 1.04:1 with a slim confidence interval of 0.97 to 1.10 and therefore did not differ from an equal ratio. Cochrane’s *Q* test revealed no significant heterogeneity between studies (*Q* = 8.14, df = 11, *p* = 0.61), which was further confirmed with a more powerful quantity *I*^2^ that established 0% of total variation across studies was due to heterogeneity instead of chance. Therefore, male and female patients are burdened by TSC at equal rates and any variance of the M:F ratio is due to statistical chance alone.

Patient ages in the whole cohort (as of October 2020) ranges between < 0 (prenatal) and 75 years of age, with median ages of 17 and 20 years old between TSC1 and TSC2 groups, respectively. The age distribution between TSC1 and TSC2 groups is very similar, with median ages of first symptom detection in both groups being at < 1 year of age with interquartile ranges of 2.25 and 2 years respectively. The standard deviation of age at first inclusion was 1.91 in the TSC1 group, and 8.01 in the TSC2 group indicating a greater variety of detection age in the latter (Additional file 1: Table S1).

### Prevalence of CHD

The overall aim of this investigation is to characterise the nature of the relationship between CHDs, NDDs and KDs in *TSC1* and *TSC2* patients. To establish the association between the three conditions, it is important to first investigate each manifestation at its individual organ level. Firstly, CHD prevalence in *TSC1* and *TSC2* patients in this cohort were determined, with 57% and 75% of patients respectively receiving a diagnosis of either single or multiple CR at some point during their lifetime, with no significant difference in rhabdomyoma prevalence between the two populations found (*z* = −1.56, *p* = 0.119) (Fig. 3A).

The location of rhabdomyoma within the heart was also compared between *TSC1* and *TSC2* patients to evaluate whether their genotype impacted the preferential location CR development within different heart structures. The position of CRs within the heart was categorised into five primary locations; interventricular septum (IVS), left ventricle (LV), right ventricle (RV), left atrium (LA) and right atrium (RA) (Fig. 3B). Cumulatively, it was found that 98% of all TSC patients with CR had at least one tumour located in either ventricle or the IVS (Fig. 3B). Despite a marked difference between the prevalence of CR in the ventricles versus the atria in all patients, no significant difference of CR prevalence in each structure was found when comparing *TSC1* vs *TSC2* hearts (Fig. 3B), implying that the tumours appear at the same rate in each heart structure no matter the genotype (IVS, *z* = −0.162, *p* = 0.873; LV, *z* = 0.695, *p* = 0.490; RV, *z* = 0.969, *p* = 0.332; LA, *z* = −1.02, *p* = 0.308; RA, *z* = 0.681, *p* = 0.497).

In addition to the structural location of CR, the number of individual tumours is also a considerable factor in the overall cardiovascular health of TSC patients. The proportion of individuals with multiple CRs as opposed to an isolated tumour stands at 86% of *TSC1* patients and 89% of *TSC2* patients with cardiac manifestations (Fig. 3C).

Overall, most patients with multiple CR had either two or three tumours, representing 43% and 65% of the patients in TSC1 and TSC2 cohorts, respectively (Fig. 3C). There was no significant difference between number of tumours observed between *TSC1* and *TSC2* patients; therefore, we hypothesise that the number of tumours follow the same distribution, no matter the genotype (1 tumour, *z* = 0.268, *p* = 0.787; 2–3 tumours, *z* = –1.10, *p* = 0.271; 4+ tumours, *z* = 1.01, *p* = 0.313). The diameter of the largest cardiac rhabdomyoma in *TSC1* and *TSC2* patients was also investigated and ranged from 5 mm up to 34 mm, with the majority (22%) ranging from 10 to 15mm (Fig. 3D). Both frequency distributions followed a normal Gaussian distribution as confirmed by a non-significant Shapiro-Wilk test result (TSC1, *W* = 0.84, df = 6, *p* = 0.099; TSC2, *W* = 0.94, df = 6, *p* = 0.61). The two distributions returned a *z*-test statistic of 1.59, and a *p* value of 0.11 indicating there was no significant difference between the largest CR diameter of *TSC1* and *TSC2* patients.

Whilst CRs are thought to be present as early as 20–30 weeks of gestation [17], in this cohort, only 43% of rhabdomyoma patients were diagnosed in utero (Fig. 3E). After birth and onset of tumour regression, once a CR has completely shrunk or remains stable in size, it is said to be ‘resolved’. An exponential graph of resolution of tumours over time (Fig. 3F) revealed an average CR resolution age of 6.8 years old, with an average resolution age of 6.7 years old in *TSC1* patients, and 7.2 years old in *TSC2* patients. However, no further tumour regression was observed in either *TSC1* or *TSC2* patients beyond 15.5 years of age, meaning that for approximately 16% of patients, CR persisted into adulthood.

#### Prevalence of NDD

**Fig. 4:**
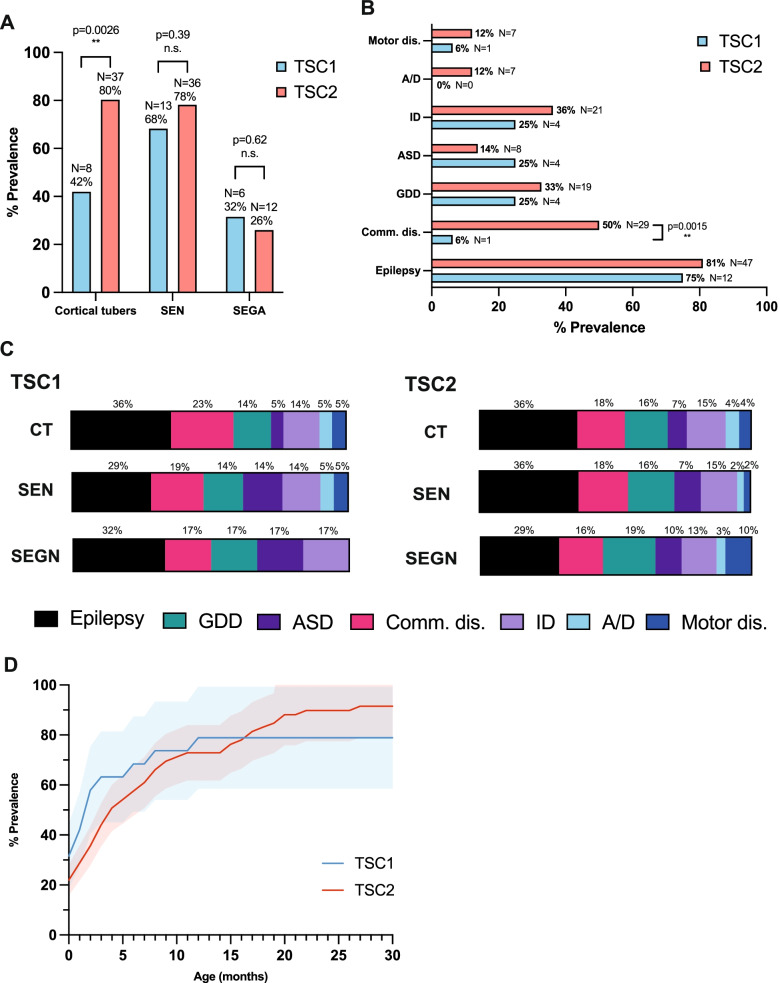
Characterisation of brain involvement in TSC1 and TSC2 patients by brain MRI and TAND assessment. **A** The prevalence of cortical tubers, subependymal nodules (SEN) and subependymal giant cell astrocytoma (SEGA) in the brains TSC1 vs TSC2 patients (TSC1, *N* = 19; TSC2, *N* = 46); **B** The prevalence of TSC-associated neuropsychiatric disorders (TAND) and epilepsy in TSC1 vs TSC2 patients; **C** TAND prevalence by brain lesion type for TSC1 vs TSC2; **D** The prevalence of brain lesions as patients age TSC1 vs TSC2 (mean ± SEM); TSC1 vs TSC2 statistically compared with *Z*-score for 2 population proportions (*p* < 0.05 significance); MRI, magnetic resonance imaging; motor dis., motor disorders; A/D, anxiety/depression; ID, intellectual disability; ASD, autism spectrum disorder; GDD, global developmental delay; comm. dis., communication disorder; ** *p* < 0.01; n.s., non-significant

In addition to structural abnormalities within the brain, often seizures and delayed developmental milestones will become apparent during infancy and childhood, potentially leading to a diagnosis of some form of the neurodevelopmental disorder (NDD) which lie under the umbrella term of TSC-associated neuropsychiatric disorders (TAND). The areas of cerebral cortical dysplasia in the form of cortical tubers (CT) were prevalent in 42% and 80% of *TSC1* and *TSC2* patients, respectively (Fig. 4A). By employing a *Z*-score test for two population proportions, this marked difference between the two groups was deemed statistically significant (*Z* = −3.0, df = 1, *p* = 0.0026). Subependymal nodules (SEN) appear deeper within the brain, affecting 68% and 78% of TSC1 and 2 patients in their respective groups, and subependymal giant cell astrocytoma (SEGA) were detected with even smaller prevalence at 32% and 26%, respectively (Fig. 4A). Neither of these lesion types differed significantly between TSC1 and TSC2 (TSC1, *Z* = 0.85, df = 1, *p* = 0.40; TSC2, *Z* = 0.49, df = 1, *p* = 0.62).

The presence of NDDs in the cohort was evaluated from results of regular TAND screening, and the comorbidity of epilepsy was also quantified. 75% of *TSC1* patients and 81% of *TSC2* patients experienced epilepsy in the form of a range of seizure types (Fig. 4B). Statistical analysis revealed that epilepsy occurs with equal prevalence between *TSC1* and *TSC2* groups (*Z* = −0.53, df = 1, *p* = 0.60). Most of the other TAND assessed such as motor disorders, anxiety, depression and global developmental delay (GDD) also showed no significant difference in prevalence between *TSC1* and *TSC2* patient groups.

Autism spectrum disorder (ASD) and intellectual disability (ID) are thought to affect around half of all TSC patients; however, within this cohort, the ASD prevalence ranged from 14 to 25% and ID prevalence from 25 to 36% (Fig. 4B), possibly a direct result of an observed assessment gap for TAND in the UK [40]. Furthermore, a significant difference was found between the presentation of communication disorders in *TSC1* and *TSC2* at a prevalence of 6% and 50%, respectively (Fig. 4B) (*Z* = −3.17, df = 1, *p* = 0.0015).

The comorbidity between different structural brain abnormalities and NDDs was explored in this cohort. Individual instances of each type of NDD were counted for each comorbidity with either CT, SEN, or SEGA and expressed as a percentage of all NDD diagnoses for each brain lesion type (Fig. 4C). Differences in proportions of separate NDDs were unremarkable when comparing by both lesion type or genotype, and appeared in almost equal proportions as the findings from Fig. 4B, most likely a result of the technicality that often brain malformations appear together, and thus, it is difficult to quantify TAND prevalence as a comorbidity of each type of lesion separately.

Finally, the age at which patients received a brain magnetic resonance imaging (MRI) screening which returned consistent with TSC-associated brain abnormalities was graphed for both *TSC1* and *TSC2* patient groups (Fig. 4D). Both graphs of brain lesion prevalence within the cohort follow a logarithmic curve, with a plateau indicating the final prevalence, settling at 79% prevalence in the *TSC1* patient group and 92% in the *TSC2* patient group. The mean age of brain lesion detection varied slightly between the groups, with *TSC1* patients on average receiving a diagnosis at 2.5 months old, which was extended to 6.4 months old for *TSC2* patients. Both models were assessed for normal Gaussian distribution with Shapiro-Wilk test, both of which demonstrated non-normal distribution (TSC1, *W* = 0.62, df = 30, *p* < 0.0001; TSC2, *W* = 0.86, df = 30, *p* = 0.0008. A two-tailed Wilcoxon matched-pairs signed-rank test was performed and the outcome revealed no significant difference between the brain abnormality detection pattern observed between *TSC1* and *TSC2* groups (*W* = 24, df = 30, *p* = 0.82).

### Prevalence of KD

**Fig. 5:**
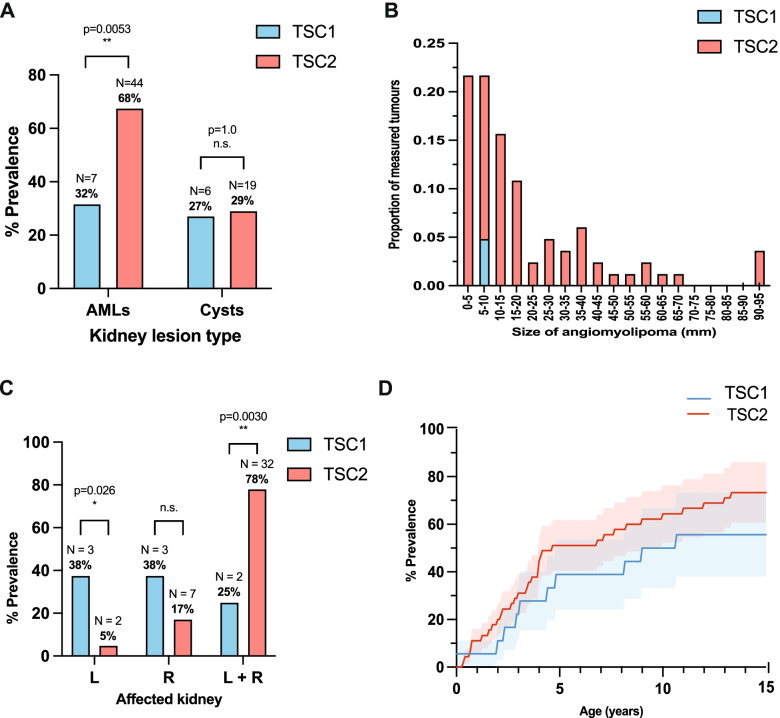
Characterisation of renal involvement in TSC1 and TSC2 patients by abdominal MRI and USS. **A** The prevalence of angiomyolipoma (AML) and cysts in the kidneys of TSC1 vs TSC2 patients (TSC1, *N*=21; TSC2, *N*=65); **B** The size of AML (mm) as a proportion of total measured tumours (TSC1, *N*=4; TSC2, *N*=68); **C** The prevalence of kidney lesions in either left kidney (L), right kidney (R) or both kidneys (L + R) in TSC1 vs TSC2; L and R compared with Fisher’s exact test (*p* < 0.05 significance); **D** The prevalence of kidney lesions as patients age TSC1 vs TSC2 (mean ± SEM); TSC1 vs TSC2 statistically compared with *Z*-score for 2 population proportions (*p* < 0.05 significance); MRI, magnetic resonance imaging; USS, ultrasound; * *p* < 0.05; ** *p* < 0.01; n.s., non-significant

**Fig. 6:**
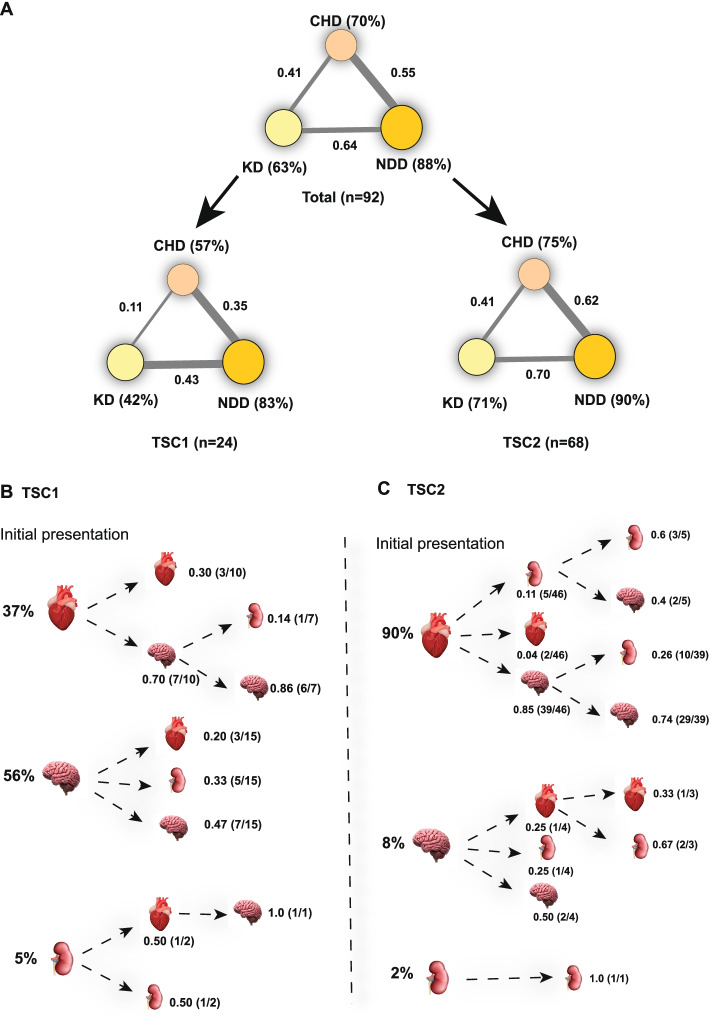
The frequency and co-occurrence of TSC manifestations and lifetime trajectory of organ involvement between CHD, NDD and KD in TSC1 and TSC2 patients. **A** Lifetime prevalence and degree of co-morbidity between CHD, NDD and KD in all patients combined, TSC1 patients and TSC2 patients. The size of circles is the proportional to the prevalence of each organ manifestation labelled in brackets, and the width of connecting lines is proportional to the degree of relative comorbidity between disorders; **B** The probability trajectory of lifetime organ involvement in TSC1 patients from point of initial presentation. Initial presentation at the base of the tree occurs in either the heart, brain or kidney and branches to other organs as indicated by transition probability on a branch; **C** The probability trajectory of lifetime organ involvement in TSC2 patients from point of initial presentation. CHD, congenital heart disorder; NDD, neurodevelopmental disorder; RD, renal disease

Unlike CRs and brain malformations, detection of kidney lesions is very rare at initial presentation [14]. A stark difference was observed between the prevalence of AMLs between *TSC1* and *TSC2* groups at 32% and 68%, respectively (Fig. 5A). Statistical quantification of this difference with the *Z*-score test for two population proportions confirmed statistical significance between these groups (*Z* = −2.9, df = 1, p = 0.0035). In contrast, this same difference is not observed in the case of renal cysts, where prevalence amongst *TSC1* and *TSC2* patients is almost equal at 27% and 29%, respectively (Fig. 6A), an insignificant difference at *p* < 0.05 (*Z* = −0.18, df = 1, *p* = 0.86).

The diameter of the largest AML in each patient was extracted from renal imaging data and categorised into small groups at 5mm increments to evaluate the distribution of tumour sizes amongst the cohort (Fig. 5B). Across all patients, an individuals’ largest observed AML measured 10 mm or smaller in 44% of cases, with an even split between tumours ≤5mm and 5–10mm in diameter. All four *TSC1* patients with recorded AML sizes also fell within the 5–10mm category. The median largest AML size across patients was determined as 15 mm with an interquartile range of 25 mm.

Renal lesion localisation across both kidneys was investigated. *TSC2* patients demonstrated a pronounced localisation within both kidneys concurrently (78%), where in contrast only a quarter of investigated *TSC1* patients had lesions within both kidneys at any one time (Fig. 5C). Statistical testing with a *Z*-score test for two population proportions revealed that this difference was indeed statistically significant (*Z* = −3.0, df = 1, *p* = 0.0030). Furthermore, lesion presence had a significantly higher degree of localisation to the left kidney in *TSC1* patients than *TSC2* patients as confirmed with Fisher’s exact test (*p* = 0.026); however, this was not the case within the right kidney where instead lesions had an equal prevalence across both groups (*p* = 0.33).

Finally, the age at which patients received a USS or MRI screening which exhibited consistency with TSC-associated renal abnormalities was graphed for both *TSC1* and *TSC2* patient groups (Fig. 5D). The choice of imaging technique reflected clinical practise and pragmatic resource availability. Patients were frequently screened with ultrasound (USS), and MRI would be requested for confirmation of growing AML. Both time graphs of kidney lesion prevalence within the cohort follow a logarithmic curve, with a plateau indicating the final prevalence of all kidney lesions within each group, settling at 56% prevalence in the *TSC1* patient group and 73% in the *TSC2* patient group. The mean age of kidney lesion detection was identical between the groups, with both *TSC1* and *TSC2* patients on average receiving a diagnosis of renal involvement at 4.7 years of age. Both models were then assessed for normal Gaussian distribution using Shapiro-Wilk test, both of which demonstrated non-normal distribution (TSC1, *W* = 0.87, df = 161, *p* < 0.0001; TSC2, *W* = 0.87, df = 161, *p* < 0.0001). A two-tailed Wilcoxon matched-pairs signed-rank test was performed and the outcome revealed a significant difference between the kidney lesion detection pattern observed between *TSC1* and *TSC2* groups (*W* = 13019, df = 161, *p* < 0.0001). A median difference of 12.2% prevalence was observed between TSC1 and TSC2 groups at any one point in time, with the prevalence of kidney lesions within the TSC1 group consistently trailing behind those in their counterpart genotype throughout life.

### Co-occurrence of CHD, NDD and KD

With organ system involvement of TSC having been characterised for this cohort on a separate basis, it is now crucial to consider the quintessence of TSC as a multisystem disorder where disease manifests simultaneously in parallel systems throughout the whole body. Firstly, the prevalence and co-occurrence of organ manifestations of TSC were calculated for CHD, NDD and KD and results were separated into either a combined association for both *TSC1* and *TSC2* patients cumulatively, as well as separate genotypes (Fig. 6A). With TSC1 and TSC2 patients combined, the prevalence of organ involvement stood at 70% of patients with a CHD, 88% with an NDD and 63% with renal disorders. Separately, only 57% of *TSC1* patients exhibited some form of cardiac involvement, as opposed to 75% of *TSC2* patients, and a further *TSC1/2* variation of 42% and 71%, respectively, was observed in the case of renal involvement. The prevalence of NDDs remained consistent between both groups at 83% and 90%, respectively.

Relative co-occurrence of different organ system manifestations was determined for the group. A consistent pattern is clear across both TSC1 and TSC2 patients where the order of co-occurrences from least probable to most probable in Boolean operator terms is *P*(*CHD* ∩ *KD*), *P*(*CHD* ∩ *NDD*) and *P*(*KD* ∩ *NDD*) (Additional file 1: Tables S2 and S3). Therefore, the most frequently observed co-morbidity throughout the whole dataset is the presence of one or more NDD and co-occurring renal involvement.

However, static data only holds a limited degree of predictive power. A temporal variable was added to the comorbidity data which enabled ‘tracking’ of a patients’ disease trajectory throughout their lifetime. Probability trees of disease trajectory throughout organ systems were generated for both *TSC1* patients (Fig. 6B) and *TSC2* patients (Fig. 6C). Initial organ presentation is characterised at the base of each tree. From an initial presentation at the ‘starter organ’, disease trajectory branches out to different organs with variable transitional probabilities. The most probable disease trajectory out of all possible disease outcome scenarios calculated for TSC2 patients was CHD → NDD at 0.85, with a further 0.74 probability of renal involvement detected afterward (Fig. 6C). Within the TSC1 patient group, the most probable trajectory was CHD → NDD at a probability of 0.70, with no further organ involvement likely at 0.86 probability (Fig. 6C). Altogether, this figure provides disease trajectories and probability for 18 different possible disease outcomes across both *TSC1* and *TSC2* patient groups.

## Discussion

This project marks the first empirical investigation of the statistical association between the aberrant heart, brain and kidney development in a regional cohort of tuberous sclerosis complex patients. Furthermore, this study is one of the first to provide comprehensive genotype-phenotype profiling of all three of the discussed organ presentations of *TSC1* and *TSC2* patients separately as opposed to a combined population where the genetic profile is negated. Additionally, establishing genotype-phenotype relationships and co-morbidity data for this study have made it possible to predict probable disease trajectories of TSC throughout a patient’s lifetime, which lays the groundwork of a powerful diagnostic tool for clinicians involved with the management of TSC patients. Other important findings of this study include significantly greater frequency of *TSC2* mutations, particularly frameshift variants; equal disease burden amongst male and female patients; delayed rhabdomyoma resolution in *TSC1* patients; the significantly greater prevalence of cortical tubers, communication disorders and angiomyolipoma in *TSC2* patients; most frequent co-occurrence of organ involvement between brain and kidneys; a diverse disease trajectory in *TSC2* patients; and most probable disease association trajectory of CHD → NDD → KD in *TSC2* individuals.

### Impact of genetic and sex differences on TSC pre-disposition

Past evidence has long postulated a significantly greater *TSC2* mutation yield in TSC patient cohorts as opposed to *TSC1*, with *TSC2* mutations accounting for approximately 70–90% of mutations within the analysed cohorts [2, 4–7]. The findings from this study also corroborate with these previous studies, indicating a significant majority of TSC patients with a mutation at the *TSC2* genetic locus, with a *TSC1*:*TSC2* mutation ratio of 2.8 *TSC2* patients for every *TSC1* patient, or *TSC1* and *TSC2* mutation rates amongst the cohort at 25% and 72%, respectively. Furthermore, the detection rate of TSC mutations was determined as 97%, superseding previous studies where the detection rate ranged from 82 to 94% [41–44].

Although the genetic profile of *TSC1/2* regions was determined to be a fundamental basis for TSC pre-disposition, the effect of patient sex was negligible. The male to female ratio (M:F) for this cohort was 1.19 male patients for every female patient, although statistically male and female patients were determined to be in equal proportions. Previous studies were compiled into a meta-analysis where the overall M:F range varied from 0.73 to 1.79 male patients for every female patient; however, ultimately a pooled M:F score of 1.04 was achieved across all studies. Hence, this study accords with previous observations that incidence of TSC is not linked with patient sex.

### Enhanced clinical phenotype severity in patients with TSC2 mutations

Considering such a pronounced disproportion between *TSC1* and *TSC2* mutational frequency, this is further mirrored by a frequently observed more severe disease phenotype in *TSC2* patients, which has largely been hypothesised to be a result of ascertainment bias [3–5, 41]. The results from this study were no different, with a more severe disease presentation observed in both the brain and kidneys; however, the heart was seemingly unaffected by genotype differences in this study.

Firstly, a significantly greater proportion of *TSC2* patients presented cortical tubers, almost double that observed in *TSC1*. Previous studies have highlighted the ambiguity of the role of *TSC1/2* in cortical tuber formation; however, experimentation with *TSC1/2* mutant mice have highlighted the critical role of both these genes for normal neuronal function as well as a high expression of these genes within the cortical plate and maturing neurons [45, 46]. Furthermore, the presence of cortical tubers has previously been associated with epilepsy and poorer neurodevelopmental outcome [47, 48]; however, no significant difference in epilepsy prevalence between *TSC1* and *TSC2* patients was found in this study. Nonetheless, a stark difference was observed in the prevalence of communication disorders between the groups, with *TSC2* patients once again demonstrating a poorer outcome. Interestingly, the prevalence of two NDDs remained markedly lower from the previously published literature on TSC. Autism spectrum disorder (ASD) and intellectual disability (ID) are thought to normally affect around half of all TSC patients [31]; however, within this cohort ASD prevalence ranged from 14 to 25% and ID prevalence from 25 to 36%. As discussed previously, this may be a direct consequence of a large assessment gap of TAND in the UK as published by the tuberous sclerosis registry to increase disease awareness (TOSCA) [40]. Patients with NDD are also less likely to have genetic testing due to a lack of personal need for reproduction. This group would have been excluded from this study. Furthermore, one would expect to see a considerably greater comorbidity between epilepsy and CTs alone based on previous studies [48]; however, the prevalence of epilepsy remains consistent between all three brain tumour types. This is most likely because often brain malformations appear together, and thus, it is difficult to quantify the TAND prevalence as a comorbidity of each type of lesion separately as they simply do not appear in isolation the majority of the time considering the extremely high prevalence of CTs and SEN amongst the TSC population.

Overall, the kidney presentation also varied between *TSC1* and *TSC2* patient cases. A greater prevalence of patients with AMLs was observed in the *TSC2* population, which has been well-documented in the past and postulated to be a result of Knudson’s two-hit events occurring less frequently in *TSC1* than TSC2 [37, 41]. No difference in renal cyst prevalence between the two genotypes in this study was found however, despite a previously observed trend of higher prevalence in *TSC2* likely due to mutations also impacting the polycystic kidney disease (*PDK1*) gene adjacent to *TSC2* [41, 49]. Furthermore, AMLs were typically located bilaterally in both kidneys in *TSC2* patients, as opposed to *TSC1* where lesions were localised in just one kidney 3/4 of the time.

### Prediction of organ manifestation co-occurrence and disease trajectory as a diagnostic tool

Arguably, the most important and novel outcome of this study is the statistical evaluation of the association between CHDs, NDDs and RDs. This suggests manifestations of TSC are perceived as dynamically interwoven with one another as opposed to simply being diagnosed and managed at their individual organ system level.

Previous research of non-TSC populations has identified associations between aberrant development in all three organs; however, the most well-documented link lies between co-occurrence of CHDs and NDDs. The heart and brain development is intimately related, and children with CHDs are well-known to be at increased risk of NDD likely due to altered perfusion and substrate delivery to the developing brain during early gestation [50, 51]. Furthermore, significant altered brain metabolism and microstructure as well as white matter immaturity and delay in cortical folding has been noted in CHD individuals shortly after birth [52, 53]. Contrary to this, the association between CHD and KD is not as well-established. Both mice and human studies have shown a significant overlap in genetic aetiology of CHD and kidney abnormalities which has been postulated to the conservation of developmental pathways and signalling mechanisms that regulate the heart and renal development; however, there is not a large volume of studies to support this hypothesis [54]. Moreover, abnormal neurodevelopment has been identified as a co-morbidity in patients with kidney disorders, most notably amongst paediatric chronic kidney disease (CKD) patients where common dysfunctions have been identified as ASD, ID, academic difficulties, attention problems and poor executive functioning [55–57].

Interestingly, in this study, the most frequent co-occurrence was witnessed between NDDs and KDs in both *TSC1* and *TSC2* patient groups, with NDD and CHD correlation as a close second. As this is a novel finding, there is little previous research to suggest why this may be the case in the context of TSC specifically; however, there is sufficient evidence from general population studies discussed previously to outline the possible aetiology behind this. However, as is the nature of TSC as a multi-system disease, different organ manifestations do not appear simultaneously and instead first become apparent at variable developmental stages. The progressive disease trajectory throughout the heart, brain and kidneys has remained elusive until now.

This study has developed a probability tree of all the possible disease outcome scenarios of *TSC1* and *TSC2* patients in this cohort using mathematical modelling of comorbidity and time-scale data to propose likely disease trajectories. The trajectories of *TSC2* were far more diverse than *TSC1*; however, co-occurrence of all three organ manifestations together was rare, occurring in only 7% of patients. Co-occurrence of two organ manifestations was most frequently observed, with CHD → NDD most probable amongst the group. A common disease trajectory of CHD → NDD and further devolvement to KD was observed in the *TSC2* patient group most frequently.

This study provides a foundation for disease outcome prediction, which is crucial for effective patient management strategy throughout the hospital system, especially when spanning different clinical departments. For these predictions to be improved, especially in the case of *TSC1* patients with a smaller a sample size, more TSC patient cohorts need to be analysed for disease trajectories to reflect a more accurate prediction.

### Strengths of this study

The TSC patient database from CAV UHB incorporates clinical data from a spectrum of patients from the South of Wales gathered over a 30-year period. This database integrates information from a multitude of hospital departments that provide a detailed account of a patient’s experience living with TSC which is invaluable for studies such as these that explore genotype-phenotype relationships as well as the trajectory of the disease. Furthermore, the duration of gathered data contributes to a timeline of TSC manifestations across patients’ lifetimes which is instrumental for tracking the trajectory of disease for future clinical applications. Therefore, these strengths have allowed the exploration of research avenues that have not yet been investigated before in TSC cohorts.

### Limitations of this study

Despite the strengths of this study, there are still a number of limitations. Firstly, the nature of TSC as a relatively rare disease with a prevalence of 1 in 6000 to 1 in 12,000 unfortunately renders the sample size relatively small at only 160 patients in South Wales from 1990 to 2020. Patients with mild manifestations may not present to medical professionals. More significantly, 43.5% of the original cohort size were excluded from analysis as outlined in Fig. 2 due to incomplete patient data records or lack of genetic testing. In addition, the size of the *TSC1* group was particularly small at only 24 patients; therefore, an accurate result may not be justified for this specific group. In future, analysis of larger cohorts or meta-analyses of multiple cohorts should be performed to increase the overall sample size and compare with the findings from this study.

Furthermore, the hypothesised association between CHDs, NDDs and KDs in this study is purely correlational. Further larger studies should be aimed at establishing a causal relationship between these and with other organ manifestations of TSC, directing particular interest at the implications of each manifestation during development. The liver, eye, lung and skin clinical manifestations of TSC were not the subjects of this study, hence their interaction with the studied systems could not be evaluated. The exclusion of other clinical manifestations potentially can influence the association across these three organs.

Lastly, the data collected in this study has not been compared with other patient cohorts, except for the male to female ratio. Such sources of ascertainment could include the Clinical Practice Research Datalink (CPRD) which includes 6 million active patients, of which over 300 patients with a TSC diagnosis were identified as of 2016 [58]. A further meta-analysis of TSC patients across multiple cohorts is crucial to confirm the observed trends in this study, as well as evaluate the overall statistical significance of findings.

As mentioned previously, the findings from this study have several implications for future clinical practice, particularly concerning the long-term care plan of TSC patients. To evaluate the practical applications of this, personalised medicine and targeted therapy avenues of disease management need to be explored. A likely candidate for such methods would be the use of human-induced pluripotent stem cells (iPSCs) to model the abnormal development of organs in vitro and further elucidate the pathophysiology behind this process. iPSCs can be derived from TSC patient somatic cells and differentiated into tissue-specific derivatives to model the development of different organs impacted by TSC and trial therapeutic agents e.g. mTOR inhibitors as a preventative measure that may alter disease progression [59, 60].

## Conclusion

This study offers an insightful initiative towards characterisation of the relationship between CHDs, NDDs and KDs in *TSC1* and *TSC2* patients. Specifically, analysis of disease trajectories throughout TSC patients’ lifetimes lays the groundwork for future prediction of disease outcomes from initial presentation, and future long-term studies of larger TSC cohorts should aim to improve the accuracy of these predictions. Investigation of the pathophysiological aetiology behind the correlations identified here and any future studies is also essential, and the enterprising field of iPSC technology is a likely candidate for disease modelling and a starting point for novel therapeutic agents. Many patients with multiorgan disease experience fragmented care, and we have highlighted previously the need for adequate centralised care of TSC patients that adheres to published guidelines to alleviate the high burden of illness on patients and their support network. Such research as outlined here is a unique first step towards the development of updated patient management guidelines and novel treatments, in addition to opening avenues for further understanding of the dynamic role between genetics, heart function, brain function and kidney function during early development.

## Supplementary Information

**Additional file 1: Table S1.** Age distribution of TSC patients in the cohort. **Table S2.** Disease trajectory outcome probability in TSC1 patients where initial presenting organ or ‘start point’ is known or unknown (*N* = 24). **Table S3.** Disease trajectory outcome probability in TSC2 patients where initial presenting organ or ‘start point’ is known or unknown (*N* = 68). **Figure S1.** QQ plot of Shapiro-Wilk test for normality of rhabdomyoma size distribution TSC1 vs TSC2. **Figure S2.** Residual plot of the difference between observed value and predicted value of remaining rhabdomyoma prevalence from regression trendline in TSC1 rhabdomyoma group (*N* = 6). **Figure S3.** Residual plot of the difference between observed value and predicted value of remaining rhabdomyoma prevalence from regression trendline in TSC2 rhabdomyoma group (*N* = 28). **Figure S4.** Residual plot of the difference between observed value and predicted value of brain lesion prevalence from logarithmic trendline in TSC1 brain lesion group (*N* = 19). **Figure S5.** Residual plot of the difference between observed value and predicted value of brain lesion prevalence from logarithmic trendline in TSC2 brain lesion group (*N* = 59). **Figure S6.** QQ plot of Shapiro-Wilk test for normality of brain lesion prevalence TSC1 vs TSC2. **Figure S7.** QQ plot of Shapiro-Wilk test for normality of AML size distribution TSC1 vs TSC2. **Figure S8.** Residual plot of the difference between observed value and predicted value of AML prevalence from logarithmic trendline in TSC1 group (*N* = 18). **Figure S9.** Residual plot of the difference between observed value and predicted value of AML prevalence from logarithmic trendline in TSC2 group (*N* = 45). **Figure S10.** QQ plot of Shapiro-Wilk test for normality of AML prevalence TSC1 vs TSC2.

## Data Availability

The data that supports the findings are available upon request from corresponding authors.

## Abbreviations

ADHD: Attention-deficit/hyperactivity disorder
AML: Angiomyolipoma
ASD: Autism spectrum disorder
CAV UHB: Cardiff and Vale University Health Board
CHD: Congenital heart defect
CKD: Chronic kidney disease
CPRD: Clinical Practice Research Datalink
CR: Cardiac rhabdomyoma
CT: Cortical tubers
DSM-5: Diagnostic and Statistical Manual of Mental Disorders, 5th Edition
GDD: Global developmental delay
iPSCs: Induced pluripotent stem cells
ID: Intellectual disability
INDEL: Insertion-deletion mutation
IVS: Interventricular septum
KD: Kidney disorders
LA: Left atrium
LV: Left ventricle
MRI: Magnetic resonance imaging
NDD: Neurodevelopmental disorders
NGS: Next-generation sequencing
PKD: Polycystic kidney disease
RA: Right atrium
RV: Right ventricle
SEN: Subependymal nodules
SEGA: Subependymal giant cell astrocytoma
SNV: Single-nucleotide variants
SS: Sanger sequencing
SV: Small variants
TAND: TSC-associated neuropsychiatric disorders
TSC: Tuberous sclerosis complex
USS: Ultrasound
