# Supplementary material for: The association of neurodevelopmental abnormalities, congenital heart and renal defects in a tuberous sclerosis complex patient cohort
Source: BMC Med. 2022 Apr 20;20:123. doi: 10.1186/s12916-022-02325-0 (PMC9019964; doi:10.1186/s12916-022-02325-0)
Supplement: Supplementary file 1 — Additional file 1: Table S1. Age distribution of TSC patients in the cohort. Table S2. Disease trajectory outcome probability in TSC1 patients where initial presenting organ or ‘start point’ is known or unknown (N = 24). Table S3. Disease trajectory outcome probability in TSC2 patients where initial presenting organ or ‘start point’ is known or unknown (N = 68). Figure S1. QQ plot of Shapiro-Wilk test for normality of rhabdomyoma size distribution TSC1 vs TSC2. Figure S2. Residual plot of the difference between observed value and predicted value of remaining rhabdomyoma prevalence from regression trendline in TSC1 rhabdomyoma group (N = 6). Figure S3. Residual plot of the difference between observed value and predicted value of remaining rhabdomyoma prevalence from regression trendline in TSC2 rhabdomyoma group (N = 28). Figure S4. Residual plot of the difference between observed value and predicted value of brain lesion prevalence from logarithmic trendline in TSC1 brain lesion group (N = 19). Figure S5. Residual plot of the difference between observed value and predicted value of brain lesion prevalence from logarithmic trendline in TSC2 brain lesion group (N = 59). Figure S6. QQ plot of Shapiro-Wilk test for normality of brain lesion prevalence TSC1 vs TSC2. Figure S7. QQ plot of Shapiro-Wilk test for normality of AML size distribution TSC1 vs TSC2. Figure S8. Residual plot of the difference between observed value and predicted value of AML prevalence from logarithmic trendline in TSC1 group (N = 18). Figure S9. Residual plot of the difference between observed value and predicted value of AML prevalence from logarithmic trendline in TSC2 group (N = 45). Figure S10. QQ plot of Shapiro-Wilk test for normality of AML prevalence TSC1 vs TSC2. [file 12916_2022_2325_MOESM1_ESM.docx]

**^Additional file 1^**

**Table S1:** Age distribution of TSC patients in the cohort

|  | Age range (years) (Oct ’20) | Age first at inclusion (years) | | | Duration of observation (years) | Total cumulative years of observation (years) |
| --- | --- | --- | --- | --- | --- | --- |
|  |  | Median | IQR | SD | Mean |  |
| TSC1 | <0 - 75 | <1 | 2.25 | 1.91 | 20.8 | 500 |
| TSC2 | 2 - 69 | <1 | 2.00 | 8.01 | 19.4 | 1320 |

**Table S2:** Disease trajectory outcome probability in TSC1 patients where initial presenting organ or ‘start point’ is known or unknown (N=24).


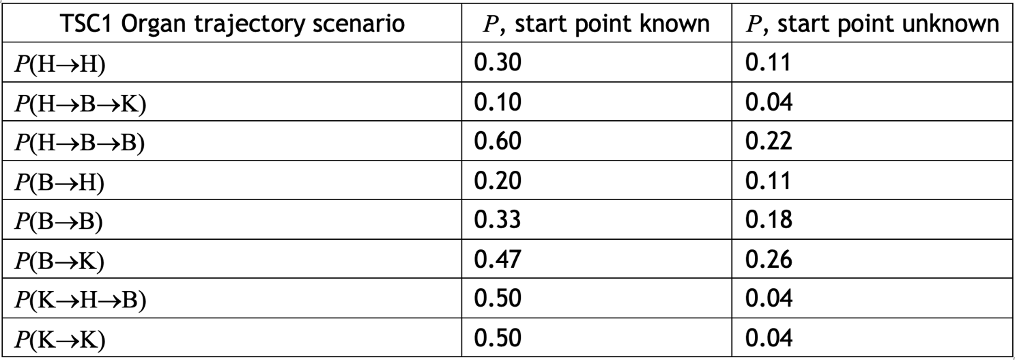


**Table S3:** Disease trajectory outcome probability in TSC2 patients where initial presenting organ or ‘start point’ is known or unknown (N=68).

**
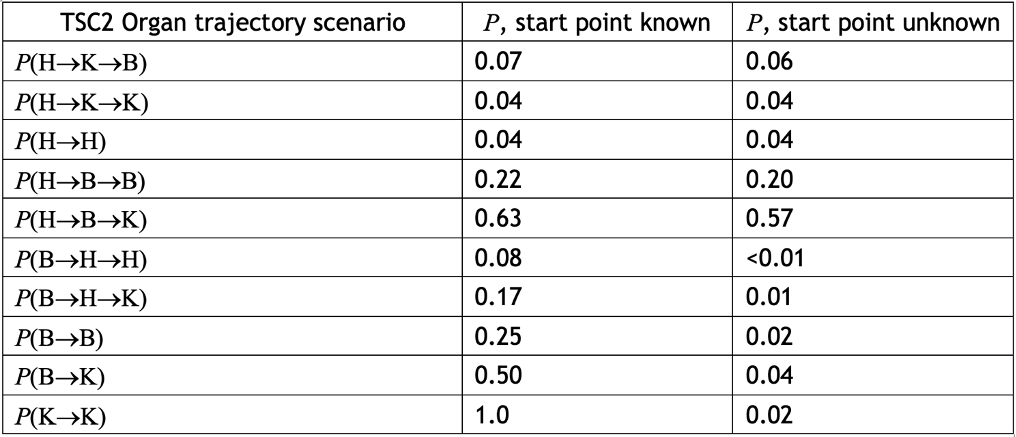
**

**Supplementary Figure And Legends:**

**
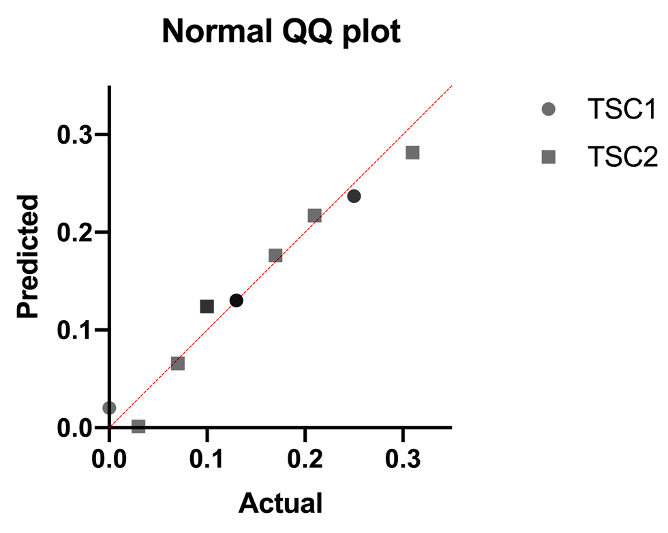
**

**Figure S1:** QQ plot of Shapiro-Wilk test for normality of rhabdomyoma size distribution TSC1 vs TSC2.

**
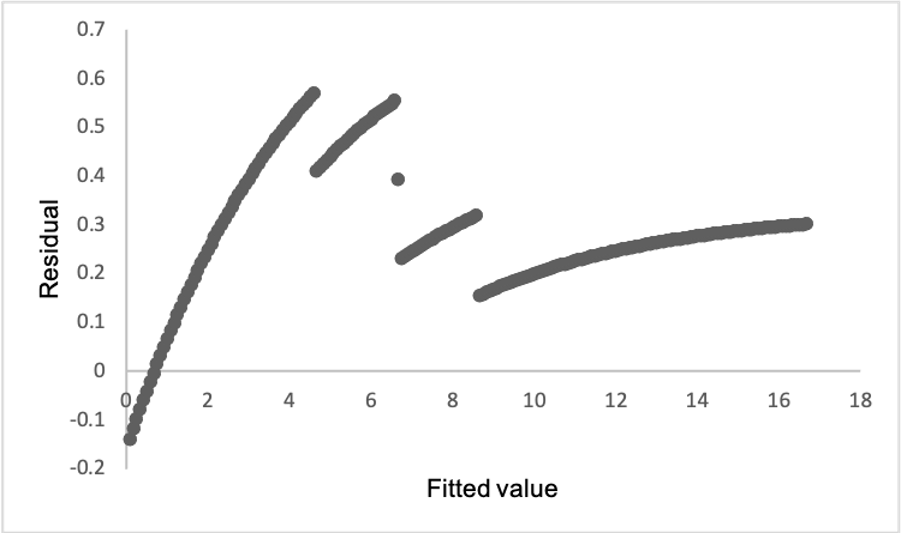
**

**Figure S2:** Residual plot of the difference between observed value and predicted value of remaining rhabdomyoma prevalence from regression trendline in TSC1 rhabdomyoma group (N=6).

**
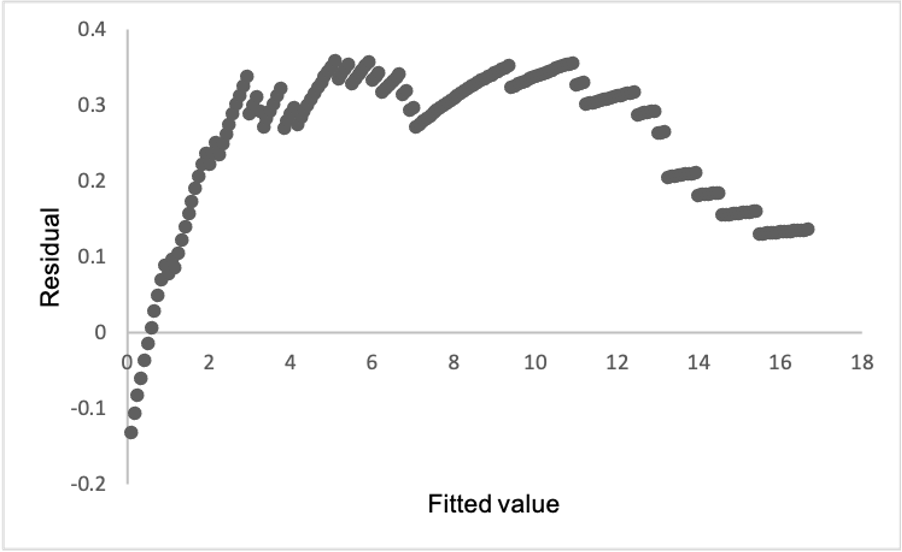
**

**Figure S3:** Residual plot of the difference between observed value and predicted value of remaining rhabdomyoma prevalence from regression trendline in TSC2 rhabdomyoma group (N=28).

**
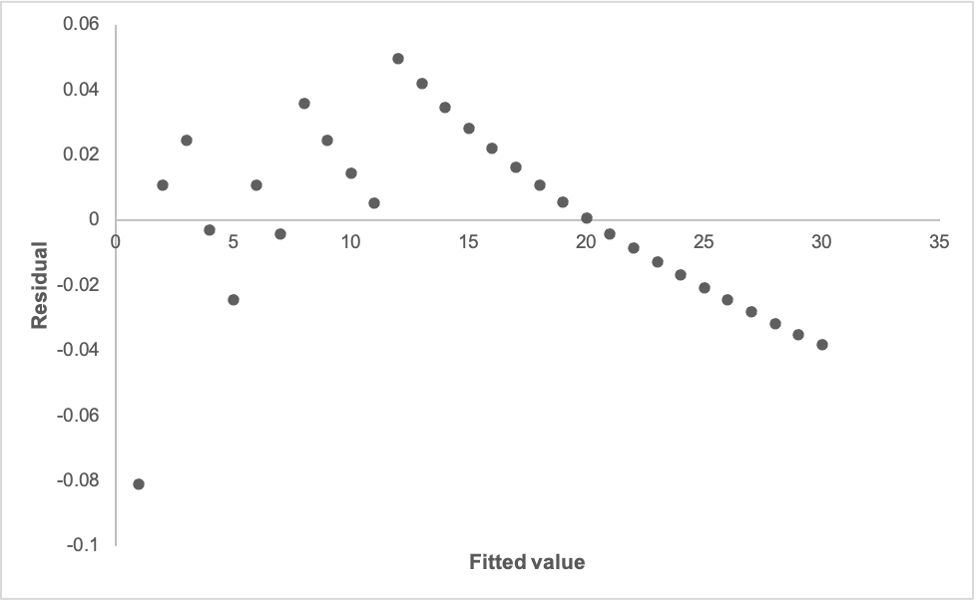
**

**Figure S4:** Residual plot of the difference between observed value and predicted value of brain lesion prevalence from logarithmic trendline in TSC1 brain lesion group (N=19).

**
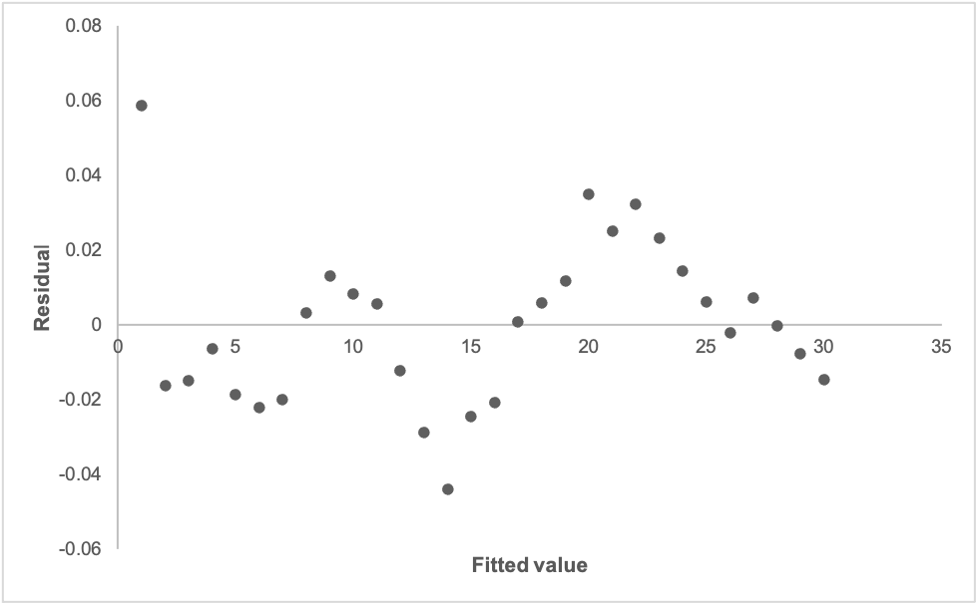
**

**Figure S5:** Residual plot of the difference between observed value and predicted value of brain lesion prevalence from logarithmic trendline in TSC2 brain lesion group (N=59).

**
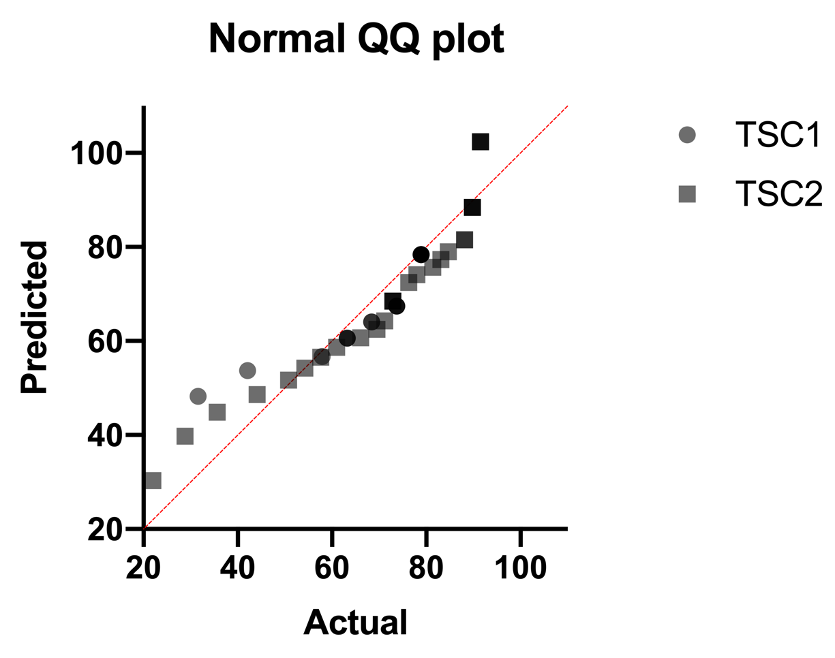
**

**Figure S6:** QQ plot of Shapiro-Wilk test for normality of brain lesion prevalence TSC1 vs TSC2.

**
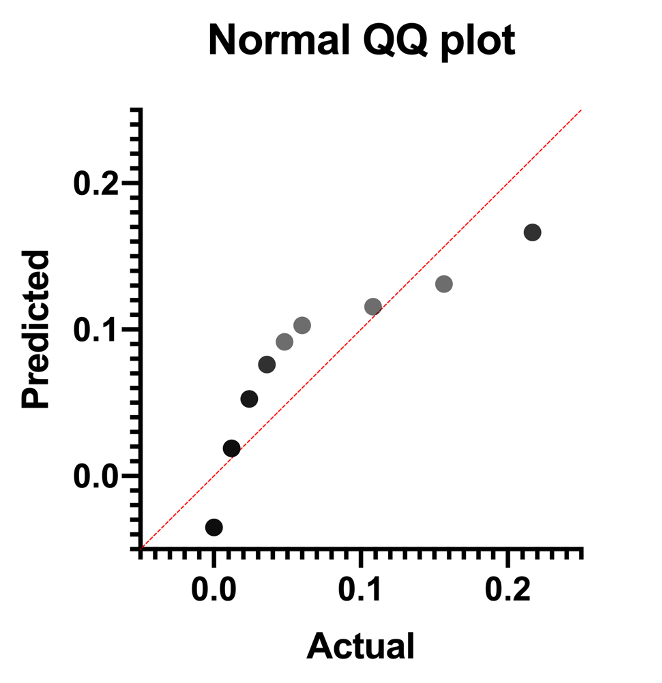
**

**Figure S7:** QQ plot of Shapiro-Wilk test for normality of AML size distribution TSC1 vs TSC2.

**
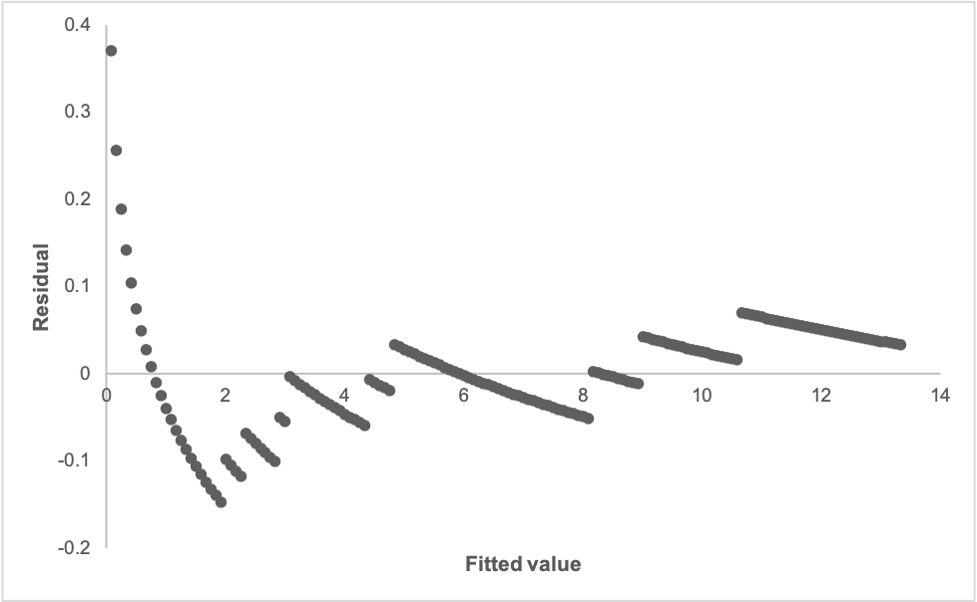
**

**Figure S8:** Residual plot of the difference between observed value and predicted value of AML prevalence from logarithmic trendline in TSC1 group (N=18).

**
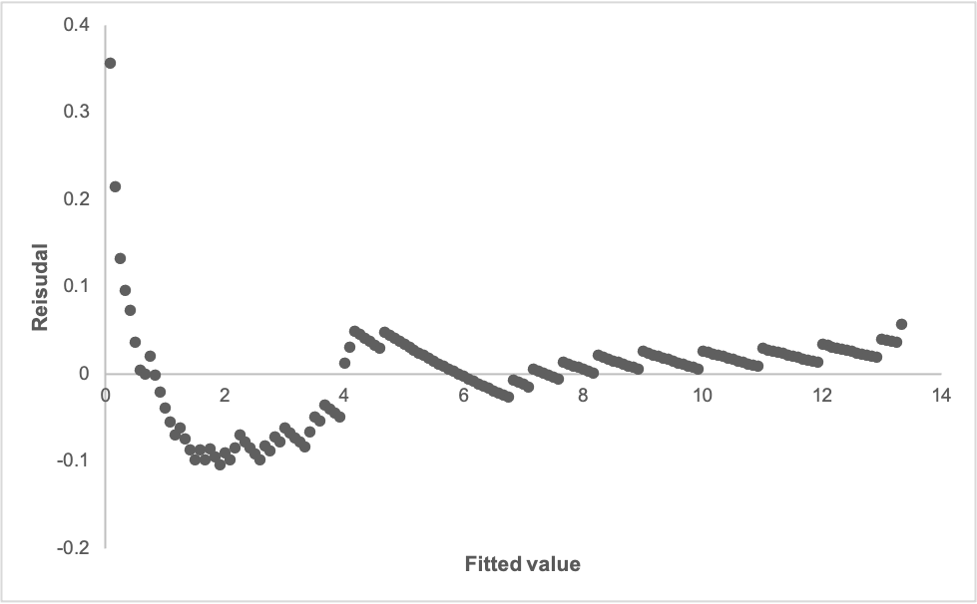
**

**Figure S9:** Residual plot of the difference between observed value and predicted value of AML prevalence from logarithmic trendline in TSC2 group (N=45).


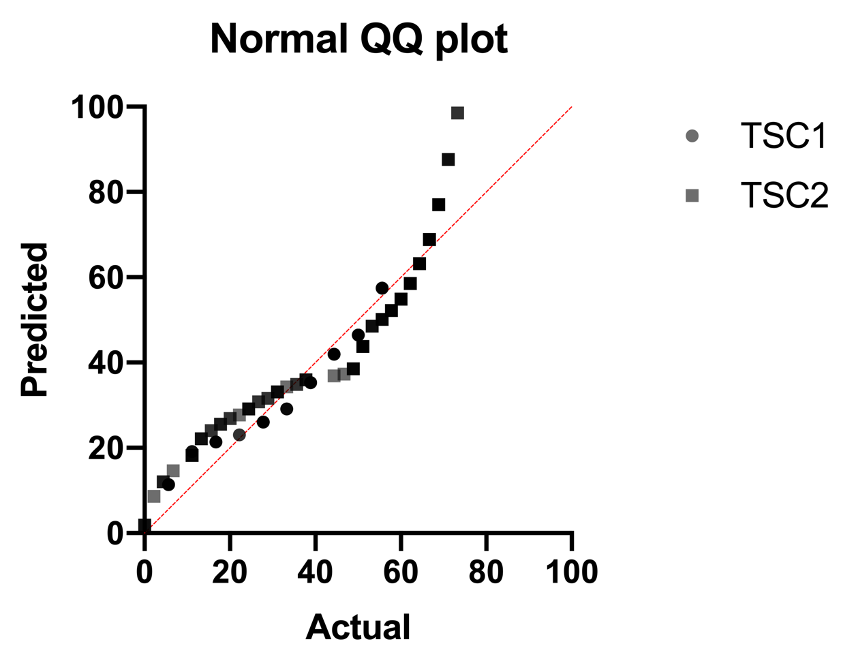


**Figure S10:** QQ plot of Shapiro-Wilk test for normality of AML prevalence TSC1 vs TSC2.
